# Microsaccades and covert attention: Evidence from a continuous, divided attention task

**DOI:** 10.16910/jemr.12.6.6

**Published:** 2019-06-28

**Authors:** Aimee E. Ryan, Brendan Keane, Guy Wallis

**Affiliations:** Centre for Sensorimotor Performance, University of Queensland, Australia

**Keywords:** Microsaccades, eye movements, eye tracking, attention, covert attention, divided-attention, visual attention

## Abstract

A substantial question in understanding expert behavior is isolating where experts look, and which aspects of their environment they process. While tracking the position of gaze provides some insight into this process, our ability to attend covertly to regions of space other than the current point of fixation, severely limits the diagnostic power of such data. Over the past decade, evidence has emerged suggesting that microscopic eye movements present during periods of fixation may be linked to the spatial distribution of covert attention, potentially offering a powerful tool for studying expert behavior. To date, the majority of studies in this field have tested the link under the constraints of a trial by trial, forced-response task. In the current study we sought to examine the effect when participants performed a continuous, divided-attention task, with the hope of bridging the gap to a range of more ecological, real-world tasks. We report various aspects of the eye movement and response data including (i) the relationship between microsaccades and drift correction, (ii) response behavior in brief time periods immediately following a microsaccade, (iii) response behavior briefly preceding a microsaccade. Analysis failed to reveal a link between task accuracy and the direction of a microsaccade. Most striking however, we found evidence for a timelocked relationship between the side of space responded to and the direction of the most recent microsaccade. The paper hence provides preliminary evidence that microsaccades may indeed be used to track the ongoing allocation of spatial attention.

## Introduction

 While it is often the case that an observer attends to the visual information their eyes are directly focussed on (1), observers also have the ability to covertly attend to information within their visual periphery (2, 3). Attending to regions in this way has been shown to improve the accuracy, speed and rate of perceptual processing (4). Attention also improves spatial resolution, sensitivity to targets within the visual periphery (5, 6, 7), and even to targets within the foveola (8).

While overt attention is relatively straightforward to track using conventional eye tracking equipment, conventional gaze tracking cannot reveal whether the observer is actually attending elsewhere, severely hampering the study of an observer’s real-time scene analysis behaviour.

Over the past decade numerous studies have reported a link between covert attention and microsaccades, a form of fixational eye movement. Microsaccades (MS) are miniature, jerky, high velocity eye movements that occur at a rate of 2-3 times per second during visual fixation (9). In the past, microsaccades were thought of as random, insignificant movements that had no impact on visual perception (10) and as such were often simply filtered out during analysis. However, advances in technology have led to a resurgence of interest in this area, and it is now generally believed that despite their size, microsaccades are more than just inconsequential eye movements (11).

Microsaccades share the same amplitude/peak velocity profile of larger saccades, but are on the lower end of the scale, with the majority of MS often classified as having an amplitude of 1-2 degrees or less (9, 10). For many years, microsaccades were widely regarded as involuntary (12), however a growing body of research suggests that this is incorrect. Expert observers (13) and relatively untrained human and monkey observers (12), have been shown to be able to actively control the production of microsaccades.

Theories regarding the function of microsaccades fall broadly into four camps: (i) microsaccades are involved in correcting for ocular drift (14, 15); (ii) microsaccades aid in the prevention of image fading due to neural adaptation when fixating (9, 16, 17); (iii) microsaccades work to improve fine spatial vision (e.g. 18); or (iv) microsaccades are linked to attention. While the latter theory was originally a matter of spirited debate (e.g. 19, 20), it is now widely accepted that under appropriate conditions, shifts in attention may be reflected in changes in microsaccade direction and rate, such as a decreased rate with temporally predictable target onsets (21), or in tasks requiring greater cognitive demand (e.g. 22, 23, 24). This, in turn, is associated with changes in behaviour, such as improved target discrimination and reduced reaction times (20, 21, 23, 25, 26, 27, 28). It would be fair to say, however, that this relationship is complex, and not absolute. For example, perceptual enhancement caused by shifts in covert attention, occur even in the absence of microsaccades (8). Nonetheless, if microsaccades are associated with shifts in covert attention, they have the potential to become an invaluable tool for tracking the spatial distribution of covert attention (29).

The idea that attention and eye movements are linked is in fact not new. It can, in part, be traced back to the premotor theory of attention, which suggests oculomotor and covert attention systems are driven by the same neural systems, and consequently, are intrinsically linked (30). One of the main postulates of this theory is that a shift of attention will precede any eye movement to a target location, and enhance vision at the target site (7, 31). In fixation tasks, these saccades are able to be suppressed (32), however it is conceivable that a trace of this attention shift is reflected in microsaccadic activity. 

In addition to their broadly similar physical characteristics, saccades and microsaccades also produce similar underlying patterns of neural activation, such as within the superior colliculus, V4, and Frontal Eye Fields (33, 34, 35). These areas are also commonly activated with the control of visual attention. Neurons in these areas have been found to show enhancement pre-microsaccade (36), and post-microsaccade (37) in instances where the upcoming microsaccade direction and stimulus location are aligned. Conversely, suppression is seen in situations where upcoming microsaccade direction and stimulus location are misaligned, as well as immediately post-microsaccade, either aligned, or misaligned (33, 36). Evidence for this neural modulation has been found in far peripheral receptive fields, where one of these micro movements can halve the response gain of a neuron responding to a location >40 degrees in eccentricity when it is directed away from, rather than towards the corresponding receptive field location (36). Response gain enhancement is regarded as being indicative of attentional allocation (38). Therefore, if a target is presented within the short time period (<50ms) pre-microsaccade, participants appear to be better at target discrimination on trials where microsaccade direction and cue are congruent (36).

This background brings us to the purpose of the present study. The vast majority of previous papers on the topic have reported a link between microsaccades and attention under the constraints of a trial by trial, forced-response task. However, presumably, if there is a link this relationship should extend to other, more ecological tasks. In the current study, we considered whether accuracy in a continuous, divided-attention task, could be predicted from microsaccade activity. Specifically, whether a microsaccade directed to a specific hemifield of visual space would precede a response to stimuli located in the ipsilateral as opposed to contralateral field.

## Methods

### Participants

Sixteen participants aged 18 to 26 (11 female, 5 male) were recruited from an undergraduate population at the University of Queensland. Participants were reimbursed $15 for their hour of participation. Participants had normal or corrected-to-normal vision. The study received ethical clearance from the University of Queensland Medical Research Ethics Committee.

### Apparatus

Participants were seated 60cm in front of a computer monitor (600 x 340 mm, resolution = 1080 x 1920, 60Hz). Eye movements were monitored using an Eyelink 1000+ eye tracker (SR-Research) and recorded at a sample rate of 500 Hz. The height of the chair was adjusted to ensure that the participants’ eye height matched the centre of the screen.

### Task

The task required participants to maintain the position of two vertically moving white lines within the green zone of the indicators on screen. 

**Figure 1.**
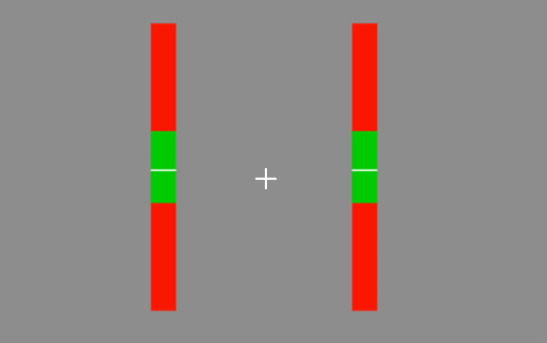
Example of task display: participants were required to fixate on the central cross. The white lines moved up and down within the indicator bars. Participants were required to maintain their position within the green zone.

Specifically, participants were instructed to maintain the white line as close to the centre of the green zone as possible. Participants were also required to maintain fixation on the central fixation cross for the duration of the trial. An example of the screen display appears in Figure 1. 

Participants controlled the white lines via key presses on the computer keyboard. The A key (up) and Z key (down) controlled the left hand indicator. The K key (up) and M key (down) controlled the white line on the right hand indicator. Pressing any of these keys once would cause the corresponding line to accelerate in the direction of the key press. The position of the indicators on the screen were at a distance of 350 pixels either side of the screen’s centre. This corresponds to a visual angle of approximately 13.1 degrees, similar to that of Engbert and Kliegl (25) who presented their peripheral targets at 12.9 DVA. The lines were injected with random accelerations throughout the trial to force the participants to closely monitor the indicator bars. The effect of these accelerations was to alter the current movement velocity by 5 pixels per second in a random direction (upward or downward). The accelerations occurred randomly but on average at a rate of once every two seconds.

Participants completed three practice trials before moving on to the main task. They then completed 24 trials, each lasting 30 seconds. A break occurred between each trial (length determined by individual participants needs), and a one-minute break was also enforced between each block.

### Measures

*Data and Statistical Analysis:* Unless otherwise stated, data analysis was performed using MATLAB (r2015a, Mathworks, Inc ©), or with IBM© SPSS Statistics 25 package, accessed via the university network.

*Performance: *Performance was assessed as the RMS pixel-wise vertical deviation of the white line from the centre of the green zone.

*Microsaccade Analysis: *Microsaccades were analysed using a modified version of the MATLAB script written by Alexander Pastukhov, itself based on the algorithm described in Engbert and Kliegl (25). This function extracts the microsaccades that were recorded by our eye tracker and records details on fixations, saccades, blinks as well as microsaccades. Any microsaccades occurring within one second of the end of the trial were excluded from analysis.

**Figure 2.**
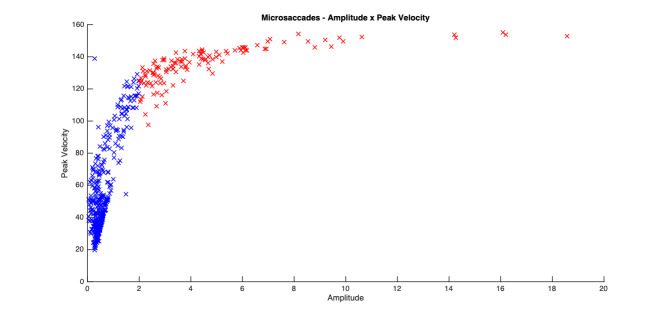
Blue – filtered microsaccades (<2 degrees), Red – unfiltered microsaccades. The figure shows that for this participant, the majority of microsaccade amplitudes are below 1DVA (M = 0.625), and all below 2DVA (the maximum threshold).

 The experimental design of the current study is clearly different from those typically employed in microsaccade studies. Despite our instructions to maintain fixation participants did occasionally break fixation. For that reason, saccades larger than 2 degrees in amplitude were excluded from further analysis (c.f. 9). Figure 2 shows a participant’s amplitude (DVA) x peak velocity graph of all saccadic events over 24 trials. The red data points are the saccades which exceeded the 2 deg. threshold. The blue data points show the microsaccades that remained after the filtering process.

*Accuracy: *Participants were told to keep the white lines as close to the centre of the green zones as possible. If the white line was below the centre point of the green zone on either indicator bar, a correct response was classed as an upwards key press, and an incorrect response as a downwards key press. Similarly, if the white line was above the centre of the green zone, a correct response corresponded to a downward key press, and an incorrect response was an upwards key press. 

*Responses*: The timing of each response was calibrated relative to the most recent microsaccade. All data were collapsed over the left and right indicator bars, giving the following response conditions:

If the first key response following a microsaccade was on the indicator bar on the same side as the direction of the microsaccade (i.e. microsaccade to the left, first key response on left), and was a correct response, this was classed as a correctSAME response. If the first key response was on the same side as the direction of the microsaccade, and was incorrect, this was an incorrectSAME response.

Conversely, if the first key response was on the opposite side as the direction of the microsaccade (i.e. microsaccade to the left, first key response on right), and was a correct response, this was classed as a correctDIFF response. If the first key response was on the opposite side as the direction of the microsaccade and was an incorrect response, this was classed as an incorrectDIFF response. 

If a key press occurred at the exact same moment on the left and right (within 20ms), this data and the corresponding microsaccade were excluded from analysis.

## Results

### Drift Correction

As stated in the introduction, some researchers have argued that the primary role of microsaccades is to correct errors in fixation caused by drift. We looked at whether there was evidence to support this hypothesis. To do this, we compared the total number of microsaccades that were directed towards the centre fixation point, with the total directed away from centre, regardless of whether they were accompanied by a key response or not.

A paired-samples t-test was used to determine whether there was a statistically significant mean difference between the percentage of microsaccades towards fixation compared with percentage of microsaccades away from fixation. Analysis revealed no significant relationship t(12) = -1.808, p = .096. Note that the result was close to the 0.05 threshold but because there was a greater proportion of microsaccades away from fixation (M = .52, SD = .04) compared to towards fixation (M = .48, SD = .04). If microsaccades are solely a response to drift from fixation, we would have expected to see more microsaccades towards rather than away from fixation. We failed to find evidence to support this hypothesis. Indeed, if anything, the trend was in the opposite direction.

### Characterising responses

#### Response time

**Figure 3.**
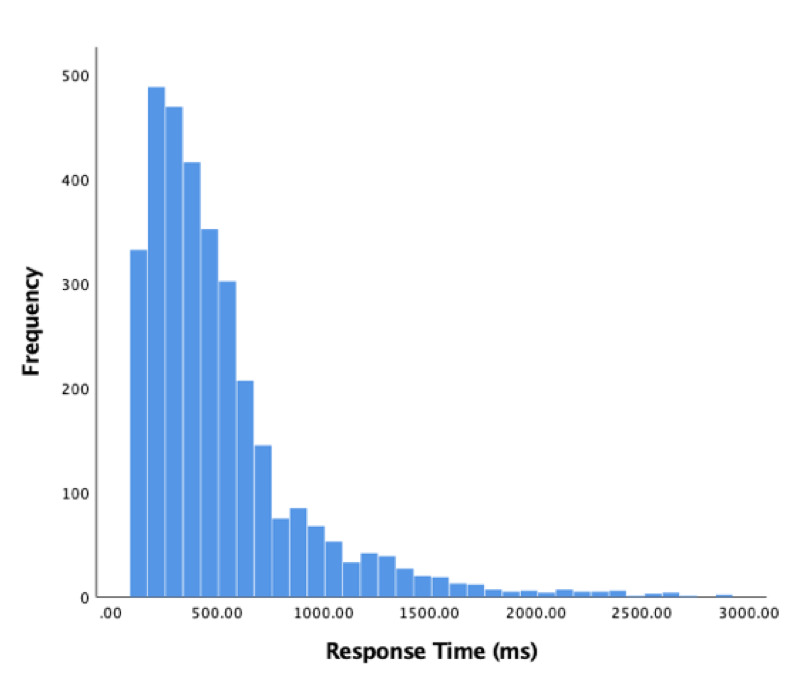
Histogram of response times (acceleration to key response) in milliseconds, 120 ms limit.

We begin analysis of our participants’ behaviour by measuring how long it took for them to respond to a sudden acceleration. Responses less than 120ms were excluded from analysis. Data was non-normally distributed; therefore, the median was used rather than mean. Of 3253 valid responses, the median response time was 400ms (SD = 386.74). A histogram of these results appears in Figure 3 below.

#### Line locus at moment of response 

**Figure 4.**
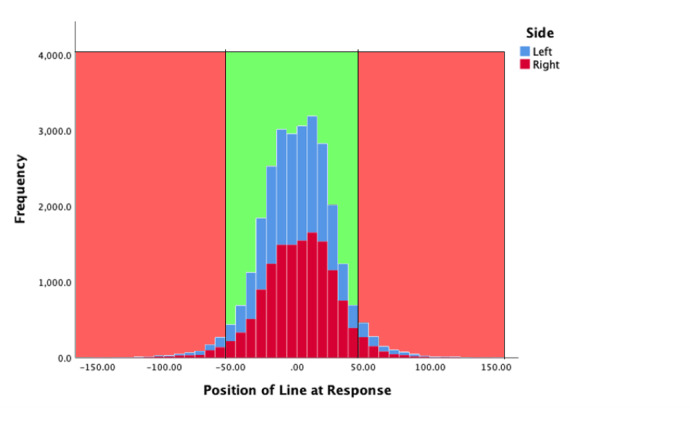
Y position of line at the moment observers pressed a response key, for both left (blue) and right (red) key presses. The light green zone corresponds to the extent of the central green zone seen by participants.

Figure 4 summarises the location of the line at the moment of a participant’s key press. In the graph zero corresponds to the middle point of the target zone (TZ). The TZ was 100 pixels tall and therefore +/- 50 mark the limits of the TZ (shown in green). There were more responses made to the left-hand bar in this case, but the distribution was similar. The mean position of the white line (yPos) when the observer pressed the key was -6.597 (SD = 27.48) and 1.8208 (SD = 27.933) for the left, and right, indicator bars respectively.

### Microsaccades and Attention

Unlike a conventional cueing paradigm, in our task the participant is free to allocate attention as they see fit. A significant assumption of our paper, therefore, is that we can intuit where participants chose to allocate attention by tracking which of the two bars they responded to, the thinking being that attention will have been directed to that bar/side of space shortly before the response was made. Part of the motivation for this is the premotor theory of attention (30) which proposes that eye movements to a spatial location are always preceded by a shift of attention to that area. Unlike cueing studies, though, we must also allow time for the motor response that we record (button press). Our own data suggest that participants regularly took up to 500ms to respond to the changes in direction of the line and we therefore searched for the motor responses for up to 500ms after each microsaccade. 

#### Response accuracy and the direction of the preceding microsaccade.

The accuracy of first responses post-microsaccade (included any data up to 500ms post-MS) was compared to baseline accuracy level (overall accuracy for every response made throughout the trial, regardless of whether it was coupled with a MS or not). Data was averaged over all trials for each participant, and then analyses run. One extreme outlier was removed, as assessed through visual inspection of box plot. A paired samples t-test demonstrated a statistically significant difference in the proportion of correct responses following a microsaccade (*M* = 81.2%, *SD* = 11.3) compared to the baseline accuracy rate (*M* = 79.2%, *SD* = 10.7%), *t*(13) = 2.756, *p* = .016. Although only a small overall difference, these results imply that microsaccades are positively linked to task accuracy.

To characterise this effect more precisely we next divided the data into time bins. Response pairings were time-locked to the onset of a valid MS (time 0) and categorised into eleven time bins: -50 to 0ms; 0 to 50ms; 50 to 100ms; 100 to 150ms; 150 to 200ms; 200 to 250ms; 250 to 300ms; 300 to 350ms; 350 to 400ms; 400 to 450ms; 450 to 500ms. Following a microsaccade, the very first response (on either the left or right key) was included in the corresponding time bin. The raw number of microsaccade response pairings in each time bin are detailed in Table 1 below, however the following analyses were conducted on proportional data that had been collapsed over all trials for each participant.

**Table 1:**
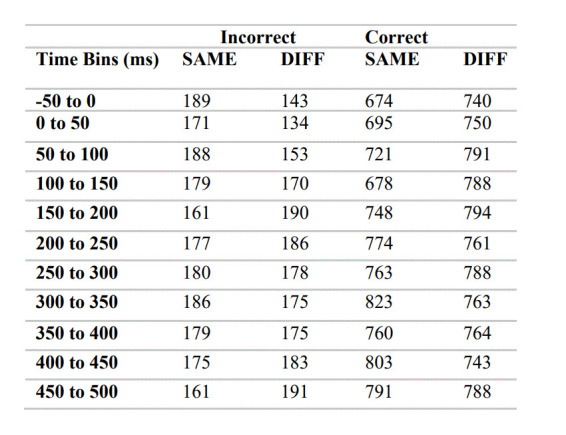
Raw number of microsaccade-response pairings included in each condition. Data was averaged for each participant over the 24 trials, prior to the following analysis.

In traditional microsaccade studies, when microsaccades are aligned with the cue location, performance is enhanced (e.g. 36). In order to perform a comparable analysis, we compared accuracy when microsaccades were on the same side (SAME), or opposite side (DIFF) to the key response made. If microsaccade direction is an indicator of attention, we may expect to see a greater proportion of correctSAME response pairings compared to correctDIFF. In other words, when the microsaccade is directed to the side that the key response was made, participants should be more likely to respond correctly.

#### Correct Responses

**Figure 5.**
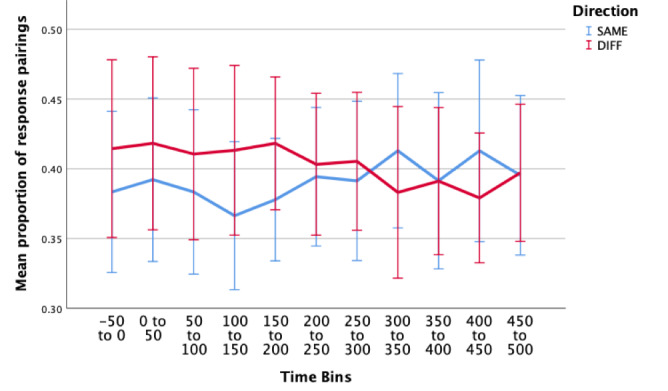
Estimated marginal means of proportion of correct response pairings (correctSAME – blue; correctDIFF - red) over multiple time periods surrounding a microsaccade. There were no significant main effects or interaction effects in this condition. Error bars: 95% CI.

A two-way repeated measures ANOVA (11 x 2) was conducted to examine the effect of time around microsaccade, and direction (SAME/DIFF) on proportion of correct response pairings (Figure 5). For all the following analyses, data was averaged over all trials for each participant. Two data sets were removed as outliers and therefore removed for analysis (studentized residuals > 3 standard deviations). One cell violated the assumption of normality (39), however we decided to run the test regardless, as ANOVAs are fairly robust to deviations from normality (see Maxwell and Delany (40) for a review). Mauchly’s test of sphericity indicated that the assumption of sphericity had been violated for the two-way interaction, χ2(54) = 128.91, p < .001.

The two-way interaction between direction and time was not statistically significant, F(4.058, 52.748) = 1.005, p = .414, ε = .406. When interpreting the main effects, there was no statistically significant differences in proportion of incorrect responses over the different time periods surrounding a microsaccade, F(4.320,56.166) = .663, p = .631, or in the proportion of responses for the different directions (SAME/DIFF), F(1,13) = .166, p = .690. 

#### Incorrect Responses

 Similarly, if attention reliably shifts with the direction of a microsaccade, there should be a greater proportion of incorrectDIFF than incorrectSAME response pairs. In other words, if the key response was made on the side opposite to that the microsaccade was directed, participants should be more likely to produce an incorrect response. 

A two-way repeated ANOVA (11 x 2) was conducted to examine the effect of time around microsaccade, and direction (SAME/DIFF) on proportion of response pairings in the incorrect condition. Data sets from two participants were removed, as there were not enough data points in each bin. There were no outliers, as assessed by examination of studentised residuals for values greater than ± 3. Three cells violated the assumption of normality (39), however we decided to run the test regardless, as ANOVAs are fairly robust to deviations from normality (see Maxwell and Delany (40) for a review). Mauchly’s test of sphericity indicated that the assumption of sphericity had been violated for the two-way interaction, χ2(54) = 127.422, p <.001. 

The two-way interaction between direction and time was not statistically significant, F(3.995, 51.933) = 1.465, p = .226, ε = .399. When interpreting the main effects, there was no statistically significant differences in proportion of incorrect responses over the different time periods surrounding a microsaccade, F(4.320,56.166) = .663, p = .631, or in the proportion of responses for the different directions (SAME/DIFF), F(1,13) = .060, p = .810. 

**Figure 6.**
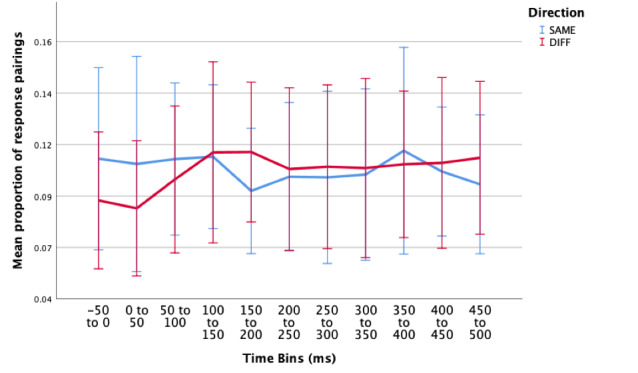
Estimated marginal means of proportion of incorrect response pairings (incorrectSAME- blue; incorrectDIFF - red) over multiple time periods surrounding a microsaccade. There were no significant main effects or interaction effects in this condition. Error bars: 95% CI

#### Laterality of response following a microsaccade, irrespective of accuracy.

One possible reason for our failure to find a link between accuracy and microsaccades is that our measure of accuracy is too coarse. It is possible that participants were acting to counter the effect of the sudden perturbation rather than being concerned with the precise location of the bar within the green zone. If there is a connection between the deployment of covert attention and the direction of a microsaccade, we reasoned that after collapsing over accuracy, there should be a greater proportion of SAME response pairings following a microsaccade, i.e. participants should be more likely to microsaccade to the bar they then respond to, rather than the opposite bar.

**Figure 7.**
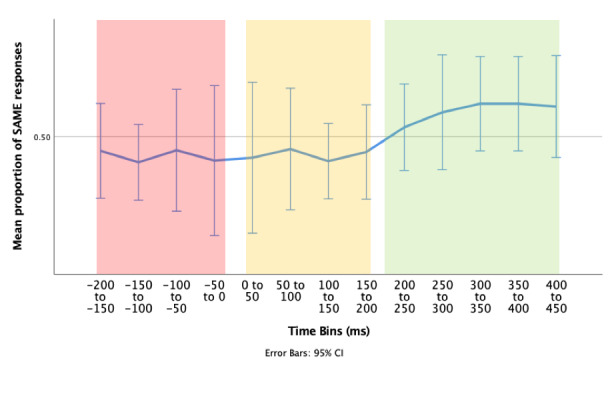
Mean proportion of SAME response pairings over different time bins (time relative to microsaccade). Pre-MS (red), Immediate Post-MS (yellow), and Post-MS motor response (green). Error bars = 95% CI.

Therefore, the data was collapsed over accuracy, and divided into three time bins (Figure 7):

1) Pre-MS (-200 to 0ms): we suggest that any responses that occur pre-microsaccade are more likely to be related to a previous shift of attention.

2) Immediate Post-MS (0 to 200ms): again, it could be suggested that any responses that occur within this time period are more likely to be related to previous attention shift, as in general, people require > 120ms for motor response. 

3) Delayed Post-MS (200ms to 450ms): we suggest that any responses that occur within this time period are most likely to be related to the current microsaccade/attention shift. This time bin includes responses that occur between 200ms and 450ms post-microsaccade (median response time ~400ms). 

Wilcoxon signed-rank tests were conducted to determine the differences in proportion of SAME response pairings between Pre-MS (1) & Delayed Post-MS (3), and, Immediate Post-MS (2) and Delayed Post-MS (3). We may expect an increased proportion of SAME response pairings in the Delayed Post-MS (3) group compared to both groups (1) and (2). All sixteen participants were included in the following analysis, as non-parametric tests are more robust to outliers.

There was a statistically significant median increase in SAME proportions from pre-MS (Mdn = 50.1%) to Delayed Post-MS (Mdn = 52.4%), z = 1.991, p = .046. There was a similar increase in proportion of SAME response pairings from Immediate Post-MS (Mdn = 50.6%), to Delayed Post-MS (Mdn = 52.4%), with this result trending towards significance, z = 1.655, p = .098.

## Discussion

In this study, we investigated the link between microsaccade direction and covert attention using a novel, continuous divided attention paradigm. Some researchers have suggested that microsaccades can be regarded as fixational corrections, whose sole purpose is to restore fixation after a period of ocular drift. We found no statistical difference in the proportion of microsaccades towards- and away from the central fixation point. Instead, if anything, we found the opposite to be true.

Previous research has demonstrated that microsaccade rate can be modulated by task requirements (e.g. 41). There is also evidence that the size and rate of microsaccades impacts the onset and secession of Troxler fading (17, 42).

In the current experiment, we report a small, yet significant difference in the accuracy rate of responses following a microsaccade compared to baseline accuracy (average accuracy rate regardless of whether response was preceded by microsaccade). The data suggest that participants were more likely to respond correctly following a microsaccade compared to baseline, although only by a small margin. This result could be taken as evidence for the idea that microsaccades help to restore, or enhance, peripheral vision. 

Finally, we tested for a relationship between the direction of a microsaccade and the side of space on which a participant responded. There were no statistically significant differences in the proportion of correctSAME vs correctDIFF response pairings, or in the proportion of incorrectSAME vs incorrectDIFF responses at any time point surrounding a microsaccade. This suggests that participants were almost equally as likely to make correct vs incorrect response on either side irrespective of the direction of the microsaccade.

However, when the data was collapsed over accuracy (now measuring the response probability) we did find evidence of an interaction between time and direction of the microsaccade plus response (SAME/DIFF). We examined the probability of response on the SAME side as a microsaccade at 3 specific time periods: 1) pre-MS, 2) immediate post-MS, 3) delayed post-MS. Even with a small sample size, a Wilcoxon signed-rank test found a significant increase in the proportion of SAME responses from Pre-MS, to Delayed Post-MS. 

As we saw earlier, the median response time following a line acceleration was 400ms. If a covert shift of attention occurs around the time of a MS, it is feasible that the benefit of this attention shift may not be present until the time for motor response is taken into account. This could explain why a greater proportion of SAME response pairings occurred in the time period 200ms to 450ms post-microsaccade. 

Although an important first step, there are a few important caveats to bear in mind when interpreting these results. As we averaged all responses, our interpretation of results assumes that each participant attended to, processed, and responded to the stimuli at a similar rate. It is conceivable that this varies across individuals and that an alternative analysis focussed on the behaviour of each individual might prove more sensitive to any effects present.

As started earlier, our results rely on the assumption that participants shift their attention to the side of screen on which they respond. To investigate this assumption in greater depth, and dispel the possibility that participants are attending to both indicator bars simultaneously (divided attention), future studies could utilise electroencephalography (EEG) recording alongside the current task. For example, differences in attentive states can be reflected in distinct oscillatory brain activity patterns. Alpha power (8-13Hz) has been found to not only be modulated globally with onset of visual stimulation, but can also react to shifts of visual attention from one hemifield to another without the movement of the eyes (covert attention). Specifically, alpha oscillations have been found to decrease in power in the hemisphere contralateral to the attended stimuli (43, 44). If correlations exist between locus of attention (as assessed through alpha power) and side of response, we could be more certain that our assumptions are valid. 

Another potential issue with our approach is that we elected to consider the first key press prior to or following a microsaccade as the participant’s response. However, it is likely that there were times when the participant analysed the bar and simply elected not to respond. For example, the participant may have attended to one of the indicator bars, but the white line was well within the target zone, and therefore no response was necessary. With our experiment design, it was not possible to be sure whether a no-response was deliberate or due to a failure to attend. 

Overall, our results suggest that after a brief processing delay post-microsaccade, participants were more likely to respond to the indicator bar that they had previously directed a microsaccade towards. This suggests that the microsaccade-covert attention link often seen in spatial cueing tasks, may also be present in the type of continuous visual attention task described in the current study. Hence, microsaccades may offer a means for studying the ongoing temporal distribution of visual spatial attention.

### Ethics and Conflict of Interest

The authors declare that the contents of the article are in agreement with the ethics described in http://biblio.unibe.ch/portale/elibrary/BOP/jemr/ethics.html and that there is no conflict of interest regarding the publication of this paper. 

### Acknowledgements

A.E.R received a PhD stipend from Boeing Defence Australia. We would like to acknowledge the significant contributions of two anonymous reviewers who provided feedback on a previous version of the manuscript.
